# Supplementary material for: Ceratopogonidae (Diptera: Nematocera) of the piedmont of the Yungas forests of Tucumán: ecology and distribution
Source: PeerJ. 2016 Nov 17;4:e2655. doi: 10.7717/peerj.2655 (PMC5119238; doi:10.7717/peerj.2655)
Supplement: Supplemental Information 1 [file peerj-04-2655-s001.pdf]

M	Season	Date	Province/State	Sites	Locality	Sites	ID	*Culicoides* sp.	*Forcipomyia* sp.	*Dasyhelea* sp.	*Atrichopogon* sp.	Others species
1	Autumn	07/07/2008	Tucumán	1	Alberdi	EL CORRALITO 1	EC1	0	1	0	0	0
1	Autumn	07/07/2008	Tucumán	2	Alberdi	EL CORRALITO 2	EC2	0	0	0	0	0
1	Autumn	07/07/2008	Tucumán	3	Alberdi	EL BADEN 1	EB1	0	0	0	0	0
1	Autumn	07/07/2008	Tucumán	4	Alberdi	EL BADEN 2	EB2	0	0	0	0	0
1	Autumn	07/07/2008	Tucumán	5	Alberdi	YANIMA 1	YA1	0	0	0	0	0
1	Autumn	07/07/2008	Tucumán	6	Alberdi	YANIMA 2	YA2	0	0	0	0	0
1	Autumn	07/07/2008	Tucumán	7	Alberdi	BAJO MARAPA 1	BM1	0	1	0	0	0
1	Autumn	07/07/2008	Tucumán	8	Alberdi	BAJO MARAPA 2	BM2	0	0	1	0	0
1	Autumn	07/07/2008	Tucumán	9	Alberdi	MARAPA CENTRAL 1	MC1	0	0	0	0	0
1	Autumn	07/07/2008	Tucumán	10	Alberdi	MARAPA CENTRAL 2	MC2	0	0	0	0	0
1	Autumn	08/07/2008	Tucumán	1	Alberdi	EL CORRALITO 1	EC1	0	0	0	0	0
1	Autumn	08/07/2008	Tucumán	2	Alberdi	EL CORRALITO 2	EC2	0	0	0	0	0
1	Autumn	08/07/2008	Tucumán	3	Alberdi	EL BADEN 1	EB1	0	0	0	0	0
1	Autumn	08/07/2008	Tucumán	4	Alberdi	EL BADEN 2	EB2	0	0	0	0	0
1	Autumn	08/07/2008	Tucumán	5	Alberdi	YANIMA 1	YA1	0	0	0	0	0
1	Autumn	08/07/2008	Tucumán	6	Alberdi	YANIMA 2	YA2	0	0	0	0	0
1	Autumn	08/07/2008	Tucumán	7	Alberdi	BAJO MARAPA 1	BM1	0	0	0	0	0
1	Autumn	08/07/2008	Tucumán	8	Alberdi	BAJO MARAPA 2	BM2	0	1	0	0	0
1	Autumn	08/07/2008	Tucumán	9	Alberdi	MARAPA CENTRAL 1	MC1	0	0	1	0	0
1	Autumn	08/07/2008	Tucumán	10	Alberdi	MARAPA CENTRAL 2	MC2	0	2	0	0	0
2	Winter	04/08/2008	Tucumán	1	Alberdi	EL CORRALITO 1	EC1	0	0	0	0	0
2	Winter	04/08/2008	Tucumán	2	Alberdi	EL CORRALITO 2	EC2	0	0	0	0	0
2	Winter	04/08/2008	Tucumán	3	Alberdi	EL BADEN 1	EB1	0	0	0	0	0
2	Winter	04/08/2008	Tucumán	4	Alberdi	EL BADEN 2	EB2	0	0	0	0	0
2	Winter	04/08/2008	Tucumán	5	Alberdi	YANIMA 1	YA1	0	1	0	0	0
2	Winter	04/08/2008	Tucumán	6	Alberdi	YANIMA 2	YA2	0	1	0	0	0
2	Winter	04/08/2008	Tucumán	7	Alberdi	BAJO MARAPA 1	BM1	0	0	0	0	0
2	Winter	04/08/2008	Tucumán	8	Alberdi	BAJO MARAPA 2	BM2	0	0	0	0	0
2	Winter	04/08/2008	Tucumán	9	Alberdi	MARAPA CENTRAL 1	MC1	0	0	0	0	0
2	Winter	04/08/2008	Tucumán	10	Alberdi	MARAPA CENTRAL 2	MC2	0	0	1	0	0

2	Winter	05/08/2008	Tucumán	1	Alberdi	EL CORRALITO 1	EC1	0	0	0	0	0
2	Winter	05/08/2008	Tucumán	2	Alberdi	EL CORRALITO 2	EC2	0	0	0	0	0
2	Winter	05/08/2008	Tucumán	3	Alberdi	EL BADEN 1	EB1	0	0	0	0	0
2	Winter	05/08/2008	Tucumán	4	Alberdi	EL BADEN 2	EB2	0	0	0	0	0
2	Winter	05/08/2008	Tucumán	5	Alberdi	YANIMA 1	YA1	0	0	0	0	0
2	Winter	05/08/2008	Tucumán	6	Alberdi	YANIMA 2	YA2	0	0	0	0	0
2	Winter	05/08/2008	Tucumán	7	Alberdi	BAJO MARAPA 1	BM1	0	0	0	0	0
2	Winter	05/08/2008	Tucumán	8	Alberdi	BAJO MARAPA 2	BM2	0	0	0	0	0
2	Winter	05/08/2008	Tucumán	9	Alberdi	MARAPA CENTRAL 1	MC1	0	0	0	0	0
2	Winter	05/08/2008	Tucumán	10	Alberdi	MARAPA CENTRAL 2	MC2	0	0	0	0	0
3	Winter	05/09/2008	Tucumán	1	Alberdi	EL CORRALITO 1	EC1	0	0	0	0	0
3	Winter	05/09/2008	Tucumán	2	Alberdi	EL CORRALITO 2	EC2	0	0	0	0	0
3	Winter	05/09/2008	Tucumán	3	Alberdi	EL BADEN 1	EB1	0	0	0	0	0
3	Winter	05/09/2008	Tucumán	4	Alberdi	EL BADEN 2	EB2	0	0	0	0	0
3	Winter	05/09/2008	Tucumán	5	Alberdi	YANIMA 1	YA1	0	0	0	0	0
3	Winter	05/09/2008	Tucumán	6	Alberdi	YANIMA 2	YA2	0	0	0	0	0
3	Winter	05/09/2008	Tucumán	7	Alberdi	BAJO MARAPA 1	BM1	0	0	0	0	0
3	Winter	05/09/2008	Tucumán	8	Alberdi	BAJO MARAPA 2	BM2	0	0	0	0	0
3	Winter	05/09/2008	Tucumán	9	Alberdi	MARAPA CENTRAL 1	MC1	0	1	0	0	0
3	Winter	05/09/2008	Tucumán	10	Alberdi	MARAPA CENTRAL 2	MC2	0	0	0	0	0
3	Winter	06/09/2008	Tucumán	1	Alberdi	EL CORRALITO 1	EC1	0	0	0	0	0
3	Winter	06/09/2008	Tucumán	2	Alberdi	EL CORRALITO 2	EC2	0	0	0	0	0
3	Winter	06/09/2008	Tucumán	3	Alberdi	EL BADEN 1	EB1	0	0	0	0	0
3	Winter	06/09/2008	Tucumán	4	Alberdi	EL BADEN 2	EB2	0	0	0	0	0
3	Winter	06/09/2008	Tucumán	5	Alberdi	YANIMA 1	YA1	0	0	0	0	0
3	Winter	06/09/2008	Tucumán	6	Alberdi	YANIMA 2	YA2	0	0	0	0	0
3	Winter	06/09/2008	Tucumán	7	Alberdi	BAJO MARAPA 1	BM1	0	0	0	0	0
3	Winter	06/09/2008	Tucumán	8	Alberdi	BAJO MARAPA 2	BM2	0	0	0	0	0
3	Winter	06/09/2008	Tucumán	9	Alberdi	MARAPA CENTRAL 1	MC1	0	0	0	0	0
3	Winter	06/09/2008	Tucumán	10	Alberdi	MARAPA CENTRAL 2	MC2	0	0	0	0	0
4	Spring	09/10/2008	Tucumán	1	Alberdi	EL CORRALITO 1	EC1	0	0	0	0	0
4	Spring	09/10/2008	Tucumán	2	Alberdi	EL CORRALITO 2	EC2	0	0	0	0	0

4	Spring	09/10/2008	Tucumán	3	Alberdi	EL BADEN 1	EB1	0	0	0	0	0
4	Spring	09/10/2008	Tucumán	4	Alberdi	EL BADEN 2	EB2	0	0	0	0	0
4	Spring	09/10/2008	Tucumán	5	Alberdi	YANIMA 1	YA1	0	0	0	0	0
4	Spring	09/10/2008	Tucumán	6	Alberdi	YANIMA 2	YA2	0	0	0	0	0
4	Spring	09/10/2008	Tucumán	7	Alberdi	BAJO MARAPA 1	BM1	0	0	0	0	0
4	Spring	09/10/2008	Tucumán	8	Alberdi	BAJO MARAPA 2	BM2	0	0	0	0	0
4	Spring	09/10/2008	Tucumán	9	Alberdi	MARAPA CENTRAL 1	MC1	0	0	0	0	0
4	Spring	09/10/2008	Tucumán	10	Alberdi	MARAPA CENTRAL 2	MC2	2	6	1	0	0
4	Spring	10/10/2008	Tucumán	1	Alberdi	EL CORRALITO 1	EC1	0	0	0	0	0
4	Spring	10/10/2008	Tucumán	2	Alberdi	EL CORRALITO 2	EC2	0	0	0	0	0
4	Spring	10/10/2008	Tucumán	3	Alberdi	EL BADEN 1	EB1	0	0	0	1	0
4	Spring	10/10/2008	Tucumán	4	Alberdi	EL BADEN 2	EB2	0	2	0	0	0
4	Spring	10/10/2008	Tucumán	5	Alberdi	YANIMA 1	YA1	0	0	0	0	0
4	Spring	10/10/2008	Tucumán	6	Alberdi	YANIMA 2	YA2	0	0	0	0	0
4	Spring	10/10/2008	Tucumán	7	Alberdi	BAJO MARAPA 1	BM1	0	0	0	0	0
4	Spring	10/10/2008	Tucumán	8	Alberdi	BAJO MARAPA 2	BM2	0	0	0	0	0
4	Spring	10/10/2008	Tucumán	9	Alberdi	MARAPA CENTRAL 1	MC1	0	0	0	0	0
4	Spring	10/10/2008	Tucumán	10	Alberdi	MARAPA CENTRAL 2	MC2	0	4	0	0	0
5	Spring	20/11/2008	Tucumán	1	Alberdi	EL CORRALITO 1	EC1	0	0	0	0	0
5	Spring	20/11/2008	Tucumán	2	Alberdi	EL CORRALITO 2	EC2	0	0	0	0	1 Alluaudomya
5	Spring	20/11/2008	Tucumán	3	Alberdi	EL BADEN 1	EB1	0	0	0	0	0
5	Spring	20/11/2008	Tucumán	4	Alberdi	EL BADEN 2	EB2	1	0	0	0	0
5	Spring	20/11/2008	Tucumán	5	Alberdi	YANIMA 1	YA1	0	0	0	0	0
5	Spring	20/11/2008	Tucumán	6	Alberdi	YANIMA 2	YA2	0	0	0	0	0
5	Spring	20/11/2008	Tucumán	7	Alberdi	BAJO MARAPA 1	BM1	0	0	0	0	0
5	Spring	20/11/2008	Tucumán	8	Alberdi	BAJO MARAPA 2	BM2	0	0	0	0	0
5	Spring	20/11/2008	Tucumán	9	Alberdi	MARAPA CENTRAL 1	MC1	0	0	0	0	0
5	Spring	20/11/2008	Tucumán	10	Alberdi	MARAPA CENTRAL 2	MC2	0	0	0	0	0
5	Spring	21/11/2008	Tucumán	1	Alberdi	EL CORRALITO 1	EC1	0	1	0	0	0
5	Spring	21/11/2008	Tucumán	2	Alberdi	EL CORRALITO 2	EC2	1	0	0	1	0
5	Spring	21/11/2008	Tucumán	3	Alberdi	EL BADEN 1	EB1	0	0	0	0	0
5	Spring	21/11/2008	Tucumán	4	Alberdi	EL BADEN 2	EB2	0	0	0	0	0

5	Spring	21/11/2008	Tucumán	5	Alberdi	YANIMA 1	YA1	0	0	0	0	0
5	Spring	21/11/2008	Tucumán	6	Alberdi	YANIMA 2	YA2	0	1	0	0	0
5	Spring	21/11/2008	Tucumán	7	Alberdi	BAJO MARAPA 1	BM1	0	0	0	0	0
5	Spring	21/11/2008	Tucumán	8	Alberdi	BAJO MARAPA 2	BM2	0	1	0	0	0
5	Spring	21/11/2008	Tucumán	9	Alberdi	MARAPA CENTRAL 1	MC1	0	0	0	0	0
5	Spring	21/11/2008	Tucumán	10	Alberdi	MARAPA CENTRAL 2	MC2	0	1	0	0	0
6	Spring	03/12/2008	Tucumán	1	Alberdi	EL CORRALITO 1	EC1	0	0	2	0	0
6	Spring	03/12/2008	Tucumán	2	Alberdi	EL CORRALITO 2	EC2	2	0	0	0	0
6	Spring	03/12/2008	Tucumán	3	Alberdi	EL BADEN 1	EB1	0	0	1	0	0
6	Spring	03/12/2008	Tucumán	4	Alberdi	EL BADEN 2	EB2	0	0	0	0	0
6	Spring	03/12/2008	Tucumán	5	Alberdi	YANIMA 1	YA1	0	0	0	0	0
6	Spring	03/12/2008	Tucumán	6	Alberdi	YANIMA 2	YA2	0	0	0	0	0
6	Spring	03/12/2008	Tucumán	7	Alberdi	BAJO MARAPA 1	BM1	1	0	0	1	0
6	Spring	03/12/2008	Tucumán	8	Alberdi	BAJO MARAPA 2	BM2	0	1	0	1	0
6	Spring	03/12/2008	Tucumán	9	Alberdi	MARAPA CENTRAL 1	MC1	0	0	0	0	0
6	Spring	03/12/2008	Tucumán	10	Alberdi	MARAPA CENTRAL 2	MC2	0	0	0	0	0
6	Spring	04/12/2008	Tucumán	1	Alberdi	EL CORRALITO 1	EC1	0	0	0	0	0
6	Spring		Tucumán	2	Alberdi	EL CORRALITO 2	EC2	0	0	0	0	0
6	Spring		Tucumán	3	Alberdi	EL BADEN 1	EB1	0	0	0	0	0
6	Spring		Tucumán	4	Alberdi	EL BADEN 2	EB2	0	0	0	0	0
6	Spring		Tucumán	5	Alberdi	YANIMA 1	YA1	0	0	0	0	0
6	Spring		Tucumán	6	Alberdi	YANIMA 2	YA2	0	0	0	0	0
6	Spring		Tucumán	7	Alberdi	BAJO MARAPA 1	BM1	0	0	0	0	0
6	Spring		Tucumán	8	Alberdi	BAJO MARAPA 2	BM2	0	0	0	0	0
6	Spring		Tucumán	9	Alberdi	MARAPA CENTRAL 1	MC1	0	0	0	0	0
6	Spring		Tucumán	10	Alberdi	MARAPA CENTRAL 2	MC2	0	0	0	0	0
7	Summer	28/01/2009	Tucumán	1	Alberdi	EL CORRALITO 1	EC1	1	14	0	2	0
7	Summer	28/01/2009	Tucumán	2	Alberdi	EL CORRALITO 2	EC2	0	0	0	0	1	Echinohelea
7	Summer	28/01/2009	Tucumán	3	Alberdi	EL BADEN 1	EB1	0	0	0	0	0
7	Summer	28/01/2009	Tucumán	4	Alberdi	EL BADEN 2	EB2	0	0	1	0	0
7	Summer	28/01/2009	Tucumán	5	Alberdi	YANIMA 1	YA1	0	10	0	0	0
7	Summer	28/01/2009	Tucumán	6	Alberdi	YANIMA 2	YA2	0	0	0	0	0

7	Summer	28/01/2009	Tucumán	7	Alberdi	BAJO MARAPA 1	BM1	0	0	2	0	0
7	Summer	28/01/2009	Tucumán	8	Alberdi	BAJO MARAPA 2	BM2	0	1	1	0	0
7	Summer	28/01/2009	Tucumán	9	Alberdi	MARAPA CENTRAL 1	MC1	0	1	1	0	0
7	Summer	28/01/2009	Tucumán	10	Alberdi	MARAPA CENTRAL 2	MC2	0	0	5	1	0
7	Summer	29/01/2009	Tucumán	1	Alberdi	EL CORRALITO 1	EC1	0	7	2	0	0
7	Summer	29/01/2009	Tucumán	2	Alberdi	EL CORRALITO 2	EC2	3	0	18	0	1 Bezzia
7	Summer	29/01/2009	Tucumán	3	Alberdi	EL BADEN 1	EB1	3	0	5	4	0
7	Summer	29/01/2009	Tucumán	4	Alberdi	EL BADEN 2	EB2	0	0	0	1	0
7	Summer	29/01/2009	Tucumán	5	Alberdi	YANIMA 1	YA1	0	8	0	0	0
7	Summer	29/01/2009	Tucumán	6	Alberdi	YANIMA 2	YA2	0	0	0	0	0
7	Summer	29/01/2009	Tucumán	7	Alberdi	BAJO MARAPA 1	BM1	0	0	0	0	0
7	Summer	29/01/2009	Tucumán	8	Alberdi	BAJO MARAPA 2	BM2	1	3	3	0	0
7	Summer	29/01/2009	Tucumán	9	Alberdi	MARAPA CENTRAL 1	MC1	0	0	0	0	0
7	Summer	29/01/2009	Tucumán	10	Alberdi	MARAPA CENTRAL 2	MC2	0	0	0	0	0
8	Summer	01/02/2009	Tucumán	1	Alberdi	EL CORRALITO 1	EC1	0	0	1	0	0
8	Summer	01/02/2009	Tucumán	2	Alberdi	EL CORRALITO 2	EC2	2	1	14	5	0
8	Summer	01/02/2009	Tucumán	3	Alberdi	EL BADEN 1	EB1	1	2	8	4	0
8	Summer	01/02/2009	Tucumán	4	Alberdi	EL BADEN 2	EB2	1	3	2	2	0
8	Summer	01/02/2009	Tucumán	5	Alberdi	YANIMA 1	YA1	0	1	0	0	0
8	Summer	01/02/2009	Tucumán	6	Alberdi	YANIMA 2	YA2	0	0	0	0	0
8	Summer	01/02/2009	Tucumán	7	Alberdi	BAJO MARAPA 1	BM1	0	3	1	0	0
8	Summer	01/02/2009	Tucumán	8	Alberdi	BAJO MARAPA 2	BM2	0	0	1	1	0
8	Summer	01/02/2009	Tucumán	9	Alberdi	MARAPA CENTRAL 1	MC1	0	0	5	0	0
8	Summer	01/02/2009	Tucumán	10	Alberdi	MARAPA CENTRAL 2	MC2	1	2	5	0	0
8	Summer	02/02/2009	Tucumán	1	Alberdi	EL CORRALITO 1	EC1	0	0	0	0	0
8	Summer	02/02/2009	Tucumán	2	Alberdi	EL CORRALITO 2	EC2	0	1	12	1	0
8	Summer	02/02/2009	Tucumán	3	Alberdi	EL BADEN 1	EB1	0	0	0	0	0
8	Summer	02/02/2009	Tucumán	4	Alberdi	EL BADEN 2	EB2	0	0	0	0	0
8	Summer	02/02/2009	Tucumán	5	Alberdi	YANIMA 1	YA1	0	0	0	0	0
8	Summer	02/02/2009	Tucumán	6	Alberdi	YANIMA 2	YA2	2	0	0	0	0
8	Summer	02/02/2009	Tucumán	7	Alberdi	BAJO MARAPA 1	BM1	0	0	0	0	0
8	Summer	02/02/2009	Tucumán	8	Alberdi	BAJO MARAPA 2	BM2	0	0	3	1	0

8	Summer	02/02/2009	Tucumán	9	Alberdi	MARAPA CENTRAL 1	MC1	0	1	2	0	0
8	Summer	02/02/2009	Tucumán	10	Alberdi	MARAPA CENTRAL 2	MC2	0	0	0	0	0
9	Summer	19/03/2009	Tucumán	1	Alberdi	EL CORRALITO 1	EC1	1	0	3	1	0
9	Summer	19/03/2009	Tucumán	2	Alberdi	EL CORRALITO 2	EC2	3	0	2	4	0
9	Summer	19/03/2009	Tucumán	3	Alberdi	EL BADEN 1	EB1	5	2	1	0	0
9	Summer	19/03/2009	Tucumán	4	Alberdi	EL BADEN 2	EB2	0	0	1	2	0
9	Summer	19/03/2009	Tucumán	5	Alberdi	YANIMA 1	YA1	0	0	0	0	0
9	Summer	19/03/2009	Tucumán	6	Alberdi	YANIMA 2	YA2	0	0	2	0	0
9	Summer	19/03/2009	Tucumán	7	Alberdi	BAJO MARAPA 1	BM1	0	0	0	0	0
9	Summer	19/03/2009	Tucumán	8	Alberdi	BAJO MARAPA 2	BM2	0	0	1	0	0
9	Summer	19/03/2009	Tucumán	9	Alberdi	MARAPA CENTRAL 1	MC1	1	0	3	0	0
9	Summer	19/03/2009	Tucumán	10	Alberdi	MARAPA CENTRAL 2	MC2	0	0	4	0	0
9	Summer	20/03/2009	Tucumán	1	Alberdi	EL CORRALITO 1	EC1	2	0	2	2	0
9	Summer	20/03/2009	Tucumán	2	Alberdi	EL CORRALITO 2	EC2	1	0	3	5	0
9	Summer	20/03/2009	Tucumán	3	Alberdi	EL BADEN 1	EB1	0	0	0	0	0
9	Summer	20/03/2009	Tucumán	4	Alberdi	EL BADEN 2	EB2	0	1	1	0	0
9	Summer	20/03/2009	Tucumán	5	Alberdi	YANIMA 1	YA1	1	0	0	0	0
9	Summer	20/03/2009	Tucumán	6	Alberdi	YANIMA 2	YA2	0	0	5	0	0
9	Summer	20/03/2009	Tucumán	7	Alberdi	BAJO MARAPA 1	BM1	0	0	1	0	0
9	Summer	20/03/2009	Tucumán	8	Alberdi	BAJO MARAPA 2	BM2	0	0	0	1	0
9	Summer	20/03/2009	Tucumán	9	Alberdi	MARAPA CENTRAL 1	MC1	0	1	3	2	0
9	Summer	20/03/2009	Tucumán	10	Alberdi	MARAPA CENTRAL 2	MC2	0	0	5	0	0
10	Autumn	22/04/2009	Tucumán	1	Alberdi	EL CORRALITO 1	EC1	0	1	4	0	0
10	Autumn	22/04/2009	Tucumán	2	Alberdi	EL CORRALITO 2	EC2	0	0	1	0	0
10	Autumn	22/04/2009	Tucumán	3	Alberdi	EL BADEN 1	EB1	0	0	0	0	0
10	Autumn	22/04/2009	Tucumán	4	Alberdi	EL BADEN 2	EB2	0	0	0	0	0
10	Autumn	22/04/2009	Tucumán	5	Alberdi	YANIMA 1	YA1	0	0	0	1	0
10	Autumn	22/04/2009	Tucumán	6	Alberdi	YANIMA 2	YA2	0	0	0	0	0
10	Autumn	22/04/2009	Tucumán	7	Alberdi	BAJO MARAPA 1	BM1	0	1	1	0	0
10	Autumn	22/04/2009	Tucumán	8	Alberdi	BAJO MARAPA 2	BM2	0	0	0	1	0
10	Autumn	22/04/2009	Tucumán	9	Alberdi	MARAPA CENTRAL 1	MC1	0	0	0	0	0
10	Autumn	22/04/2009	Tucumán	10	Alberdi	MARAPA CENTRAL 2	MC2	0	0	0	0	0

10	Autumn	23/04/2009	Tucumán	1	Alberdi	EL CORRALITO 1	EC1	0	3	6	0	0
10	Autumn	23/04/2009	Tucumán	2	Alberdi	EL CORRALITO 2	EC2	0	0	1	1	0
10	Autumn	23/04/2009	Tucumán	3	Alberdi	EL BADEN 1	EB1	1	1	1	0	0
10	Autumn	23/04/2009	Tucumán	4	Alberdi	EL BADEN 2	EB2	0	0	1	0	0
10	Autumn	23/04/2009	Tucumán	5	Alberdi	YANIMA 1	YA1	0	0	0	0	0
10	Autumn	23/04/2009	Tucumán	6	Alberdi	YANIMA 2	YA2	0	0	0	0	0
10	Autumn	23/04/2009	Tucumán	7	Alberdi	BAJO MARAPA 1	BM1	0	0	0	0	0
10	Autumn	23/04/2009	Tucumán	8	Alberdi	BAJO MARAPA 2	BM2	0	1	1	0	0
10	Autumn	23/04/2009	Tucumán	9	Alberdi	MARAPA CENTRAL 1	MC1	0	0	0	0	0
10	Autumn	23/04/2009	Tucumán	10	Alberdi	MARAPA CENTRAL 2	MC2	0	0	2	0	0
11	Autumn	02/06/2009	Tucumán	1	Alberdi	EL CORRALITO 1	EC1	0	0	1	0	0
11	Autumn	02/06/2009	Tucumán	2	Alberdi	EL CORRALITO 2	EC2	0	0	0	0	0
11	Autumn	02/06/2009	Tucumán	3	Alberdi	EL BADEN 1	EB1	0	0	1	0	0
11	Autumn	02/06/2009	Tucumán	4	Alberdi	EL BADEN 2	EB2	0	0	0	0	0
11	Autumn	02/06/2009	Tucumán	5	Alberdi	YANIMA 1	YA1	1	0	0	0	0
11	Autumn	02/06/2009	Tucumán	6	Alberdi	YANIMA 2	YA2	0	0	0	0	0
11	Autumn	02/06/2009	Tucumán	7	Alberdi	BAJO MARAPA 1	BM1	0	1	0	0	0
11	Autumn	02/06/2009	Tucumán	8	Alberdi	BAJO MARAPA 2	BM2	0	0	0	0	0
11	Autumn	02/06/2009	Tucumán	9	Alberdi	MARAPA CENTRAL 1	MC1	0	0	0	0	0
11	Autumn	02/06/2009	Tucumán	10	Alberdi	MARAPA CENTRAL 2	MC2	0	0	0	0	0
11	Autumn	03/06/2009	Tucumán	1	Alberdi	EL CORRALITO 1	EC1	0	0	0	0	0
11	Autumn	03/06/2009	Tucumán	2	Alberdi	EL CORRALITO 2	EC2	0	0	0	0	0
11	Autumn	03/06/2009	Tucumán	3	Alberdi	EL BADEN 1	EB1	0	0	0	0	0
11	Autumn	03/06/2009	Tucumán	4	Alberdi	EL BADEN 2	EB2	0	0	0	0	0
11	Autumn	03/06/2009	Tucumán	5	Alberdi	YANIMA 1	YA1	0	0	0	0	0
11	Autumn	03/06/2009	Tucumán	6	Alberdi	YANIMA 2	YA2	0	0	2	1	0
11	Autumn	03/06/2009	Tucumán	7	Alberdi	BAJO MARAPA 1	BM1	0	0	0	0	0
11	Autumn	03/06/2009	Tucumán	8	Alberdi	BAJO MARAPA 2	BM2	0	0	0	0	0
11	Autumn	03/06/2009	Tucumán	9	Alberdi	MARAPA CENTRAL 1	MC1	0	0	0	0	0
11	Autumn	03/06/2009	Tucumán	10	Alberdi	MARAPA CENTRAL 2	MC2	0	0	3	0	0
12	Winter	27/07/2009	Tucumán	1	Alberdi	EL CORRALITO 1	EC1	0	2	3	0	0
12	Winter	27/07/2009	Tucumán	2	Alberdi	EL CORRALITO 2	EC2	0	0	0	0	0

12	Winter	27/07/2009	Tucumán	3	Alberdi	EL BADEN 1	EB1	0	0	0	0	0
12	Winter	27/07/2009	Tucumán	4	Alberdi	EL BADEN 2	EB2	0	0	0	0	0
12	Winter	27/07/2009	Tucumán	5	Alberdi	YANIMA 1	YA1	0	0	0	0	0
12	Winter	27/07/2009	Tucumán	6	Alberdi	YANIMA 2	YA2	0	0	1	0	0
12	Winter	27/07/2009	Tucumán	7	Alberdi	BAJO MARAPA 1	BM1	0	0	0	0	0
12	Winter	27/07/2009	Tucumán	8	Alberdi	BAJO MARAPA 2	BM2	0	0	0	0	0
12	Winter	27/07/2009	Tucumán	9	Alberdi	MARAPA CENTRAL 1	MC1	0	0	0	0	0
12	Winter	27/07/2009	Tucumán	10	Alberdi	MARAPA CENTRAL 2	MC2	0	0	0	0	0
12	Winter	28/07/2009	Tucumán	1	Alberdi	EL CORRALITO 1	EC1	0	0	0	0	0
12	Winter	28/07/2009	Tucumán	2	Alberdi	EL CORRALITO 2	EC2	0	0	0	0	0
12	Winter	28/07/2009	Tucumán	3	Alberdi	EL BADEN 1	EB1	0	0	3	1	0
12	Winter	28/07/2009	Tucumán	4	Alberdi	EL BADEN 2	EB2	0	0	0	0	0
12	Winter	28/07/2009	Tucumán	5	Alberdi	YANIMA 1	YA1	0	0	0	0	0
12	Winter	28/07/2009	Tucumán	6	Alberdi	YANIMA 2	YA2	0	0	4	0	0
12	Winter	28/07/2009	Tucumán	7	Alberdi	BAJO MARAPA 1	BM1	0	0	0	0	0
12	Winter	28/07/2009	Tucumán	8	Alberdi	BAJO MARAPA 2	BM2	0	0	1	1	0
12	Winter	28/07/2009	Tucumán	9	Alberdi	MARAPA CENTRAL 1	MC1	0	0	0	0	0
12	Winter	28/07/2009	Tucumán	10	Alberdi	MARAPA CENTRAL 2	MC2	0	0	0	0	0
								**38**	**97**	**173**	**49**	**3**
